# Prognostic factors for trigeminocardiac reflex during cerebrovascular intervention operation

**DOI:** 10.3389/fsurg.2022.989644

**Published:** 2022-09-23

**Authors:** Zhaochu Sun, Piplu Bhuiyan, Hua Lu, Yanning Qian, Hang Xiao

**Affiliations:** ^1^ Department of Anesthesiology, The First Affiliated Hospital of Nanjing Medical University, Nanjing, China; ^2^ Department of Neurosurgery, The First Affiliated Hospital of Nanjing Medical University, Nanjing, China; ^3^ Department of Toxicology, School of Public Health, Nanjing Medical University, Nanjing, China

**Keywords:** embolization therapeutic, neurointervention, trigeminocardiac reflex, risk factor, dural arteriovenous fistula

## Abstract

**Introduction:**

Trigeminocardiac reflex (TCR) is a brainstem reflexive response of hemodynamic instability during surgery. Identification of risk factors relevant to TCR during cerebrovascular intervention procedures is helpful to efficiently prevent and treat its occurrence. The purpose of this study was to demonstrate the risk factors for Onyx embolization during cerebrovascular intervention operation so as to optimize perioperative management strategies on TCR.

**Methods:**

We performed a retrospective study on the patients with Onyx embolization under general anaesthesia over 6-years period from 2013 to 2018. 354 patients were finally eligible for inclusion, and then divided into TCR group (group T) and control group (group N). Patient characteristics, clinical diagnosis, comorbidities, lesion sites, hemodynamics changes, and complications were compared between two groups. Several multivariable regression models were applied to analyze the risk factors associated with TCR.

**Results:**

TCR occurred in 59 patients (16.7%) among 354 patients. There was no significant difference in patient characteristics between two groups (*P* > *0.05*). During DMSO/Onyx injection, HR and MAP were much lower in group T than group N (*P* < *0.01*). Notably, univariable analysis revealed that the patients with dural arteriovenous fistula (DAVF) and middle meningeal artery being affected were associated with a higher incidence of TCR (*P* < *0.01*). Furthermore, multivariable analysis showed that there was a close link of TCR with DAVF [OR = 4.12; 95% CI (1.83–10.65)] and middle meningeal artery embolization [OR = 3.90; 95% CI (1.58–9.63)]. Further stratified analysis of patients with TCR found that patients with middle meningeal artery embolization were more likely to experience hypotension during TCR episode (*P* < *0.05*). Finally, more incidence of postoperative adverse events was observed when TCR episode (*P* < *0.05*).

**Conclusion:**

We found that DAVF and middle meningeal artery embolization were independent risk factors for TCR episodes during Onyx endovascular embolization, highly likely leading to intraoperative hemodynamics fluctuations and postoperative adverse events.

## Introduction

Trigeminocardiac reflex (TCR) is a unique brain stem reflex that manifests as the sudden onset of hemodynamic perturbation in heart rate and blood pressure as a result of stimulation of any branches of the trigeminal nerve (1). There are several different subtypes of TCR, including diving reflex, Ganglion Gasseri, peripheral and central type. Cerebrovascular embolization induced TCR is one kind of central type TCR with typical brain stem reflex (2).

Inrecent years, with the development of neurointerventional technology, the treatment of Onyx embolization has gradually become an independent and important measures for the cerebrovascular malformation. This technique achieves the purpose of blood flow redistribution by blocking blood supply and occluding arteriovenous fistula (3). However, with the application of Onyx in interventional operation, the following complications and potential risks, especially TCR are common to occur. Onyx embolization induced central TCR in cerebrovascular interventional surgery usually lead to severe hemodynamic fluctuations and even cardiac arrest (4–9). Therefore, identification of risk factors underlying TCR would be helpful to establish prophylactic approaches and management strategies to efficiently prevent and treat TCR induced by Onyx embolization.

In this study, we performed a retrospective study of the patients with cerebrovascular embolization under general anaesthesia to explore the risk factors related to TCR. Aimed to quickly identify high-risk patients, prompt prevention and therapeutic measures to avoid unnecessary complications.

## Methods

This retrospective observational cohort study was approved by the Institutional Review Board (IRB) of The First Affiliated Hospital of Nanjing Medical University/Jiangsu Province Hospital (JSPH; Jiangsu, China; IRB approval number: 2020-SR-312), and registered on Chinese Clinical Trial Register (chictr.org.cn ChiCTR2000038839; October 5, 2020). Considering the retrospective design of this study, the requirement for informed consent was waived by the IRB.

### Participants and study design

This study utilised data stored and managed in the electronic medical record system of JSPH on 397 patients who were decided to perform Onyx embolization operation under general anesthesia by neurosurgeon, from January 2013 to December 2018 at a single academic institution. Patients <18 years old were excluded from the study. Similarly, patients with preoperative confusion, communication difficulties were not eligible. According to whether TCR episode during the operation, they were divided into TCR group (group T) and control group (group N). All the cases and data for the study period were screened by a group of medical record technicians in the medical informatics team who were not informed of the purpose of this study.

### Monitoring and anesthesia

All patients were performed with standardization of anesthesia induction and maintenance, intraoperative anesthetic management was performed with continuous monitoring of peripheral capillary oxygen saturation, electrocardiography (ECG), arterial blood pressure, end-tidal carbon dioxide partial pressure (PetCO_2_) and Bispectral index (BIS). Anesthesia was induced with midazolam 0.05 mg/kg, fentanyl 3 mg/kg, propofol 2 mg/kg, cisatracurium 0.15 mg/kg. Mechanical ventilation (Drager, Fabius-Plus, Germany) was used during the operation to keep PetCO_2_ at 35–45 mmHg. All patients received 1%–2% sevoflurane and 2–5 mg/kg·h propofol for anesthesia maintenance until the end of operation, and 0.1 mg/kg·h cisatracurium for muscle relaxation to half an hour before the end. The depth of anesthesia was guided by BIS <60 to prevent intraoperative awareness. If TCR occurred, it would be handled by cessation of the manipulation, and administration of vagolytic agents or adrenaline when necessary (10).

### Variables and outcomes

Perioperative variables were ascertained from patient records. The following data were recorded: patient age, sex, Body Mass Index (BMI), comorbidities, protopathy, supplying vessels, BIS value (record per 5 min during embolization), hemodynamic indexes (baseline, before embolization, embolization and end of procedure) and the use of atropine. The incidence of adverse events were also recorded including dizziness, delirium, postoperative nausea and vomiting (PONV), muscle weakness, and any other severe unexpected events (aphasia, hypopsia, etc) during operation and within the first 24 h post-operation. For the purposes of this study, TCR was defined as the sudden onset of bradycardia triggered by stimulation of the trigeminal nerve and its anatomic branches. The bradycardia is characterized by a reduction in HR of 20% or more from the baseline, and/or asystole. Change in MAP is an optional criterion for the definition of TCR (11, 12), but not included as part of the TCR definition in this study.

### Statistical analysis

All the statistical analysis was performed with SPSS 23.0 (IBM Software Inc., USA). Categorical variables were presented using numbers with percentages and were analyzed with chi-square test or Fisher's exact test, whereas the continuous variables were expressed as mean ± standard deviation, and were compared with the Student's *t*-test for unpaired samples when a normal deviation was assumed. Non-normally deviation was represented by median (M) and interquartile spacing (IQR) which analyzed by Mann-Whitney *U* test. Univariate and stepwise multivariate logistic regression analysis were performed to determine the risk factors of TCR episode. All clinically sensible covariates were included in the model. For all analysis, a *P* value of <0.05 was considered statistically significant.

## Results

**Figure 1:**
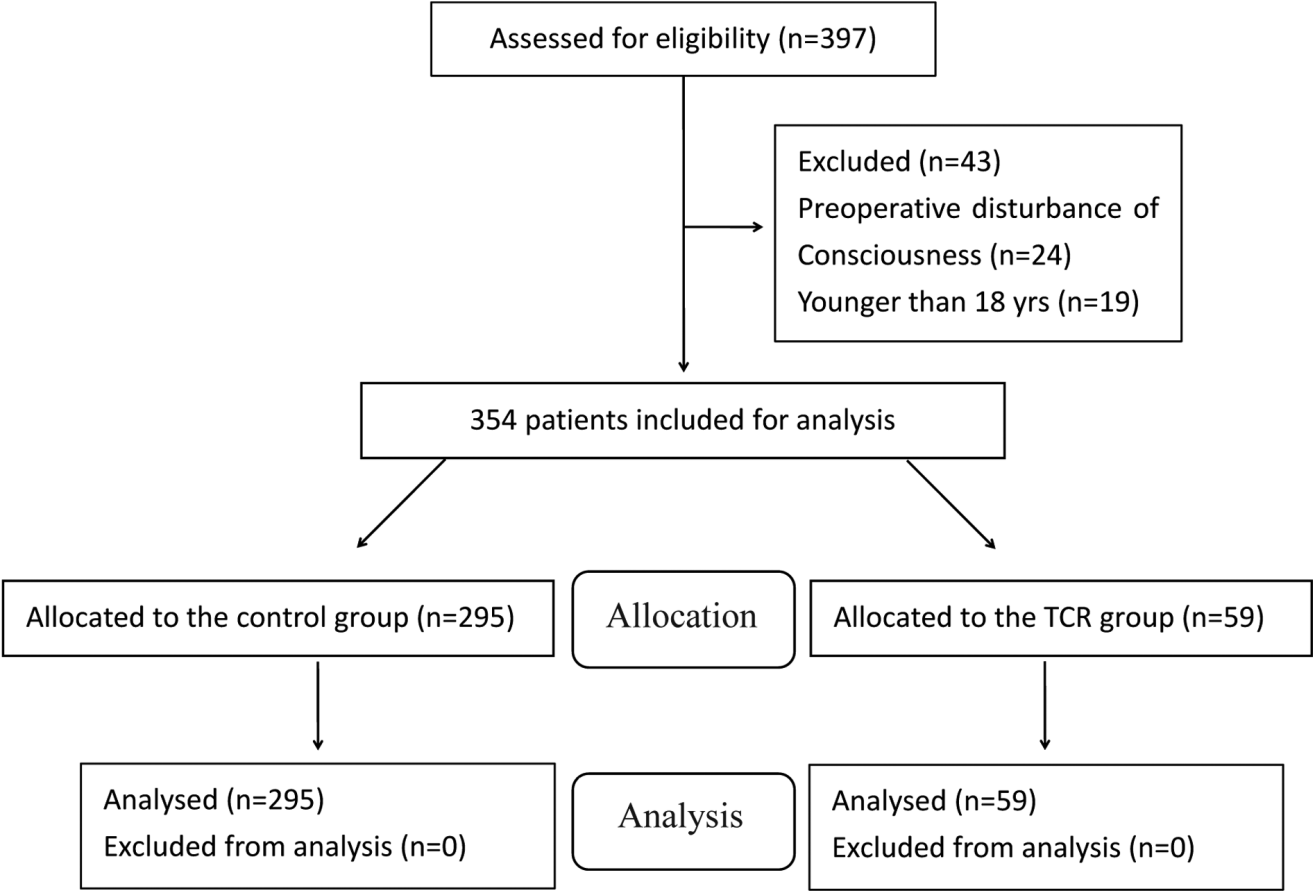
Study flow chart.

A total of 397 Chinese patients were included for participation in this study. Of these, 24 patients with preoperative disturbance of consciousness, and 19 patients younger than 18 years were excluded. Therefore, 354 patients who fulfilled the selection criteria were finally included in this study (Figure 1).

The demographic and clinical characteristics were presented in Table 1. Regarding preoperative diagnosis, 164 patients (46.3%) were diagnosed with arteriovenous malformation (AVM), 104 (29.4%) with dural arteriovenous fistula (DAVF), 32 (9.0%) with carotid-cavernous fistula (CCF), and 54 (15.3%) with intracranial tumors. There were 59 (16.7%) patients developed TCR in varying degrees, even cardiac arrest suddenly occurred in five patients along with the onset of TCR (Table 3) and resumed normal circulation after discontinuation of the surgical procedures, administration of atropine or epinephrine and even CPR. With regard to the matched variables, there was no difference between the TCR and control group including age, sex, BMI, comorbidity and BIS value. However, the protopathy, supplying vessels and the incidence of postoperative adverse events were different.

**Table 1:** Characteristics of patients exhibiting with or without TCR.

Variable	Group T (*N* = 59)	Group N (*N* = 295)	*P-*value
Age (year)	47.8 ± 12.6	45.4 ± 15.3	0.25
Gender (M/F)	41/18	177/118	0.17
BMI (Kg/m^2^)	23.7 ± 2.8	23.4 ± 2.9	0.46
Comorbidity			
Hypertension	14 (23.7)	59 (20.0)	0.52
Diabetes mellitus	5 (8.5)	20 (6.8)	0.64
Bradycardia	8 (13.6)	39 (13.2)	0.94
History of cerebral hemorrhage	18 (30.5)	111 (7.6)	0.30
Protopathy			
AVM	5 (8.5)	159 (53.9)	<0.01**
DAVF	46 (78.0)	58 (19.7)	<0.01**
CCF	4 (6.8)	28 (9.5)	0.68
Intracranial tumors	4 (6.8)	50 (16.9)	0.07
Supplying vessels			
Middle meningeal artery	43 (72.9)	47 (15.9)	<0.01**
Cerebral artery	8 (13.6)	194 (65.8)	<0.01**
Cavernous sinus	2 (3.4)	24 (8.1)	0.32
Other vessels	6 (10.2)	30 (10.2)	1.00
Bradycardia (before embolization)	20 (33.9)	81 (27.5)	0.32
BIS value	47.4 ± 6.1	46.0 ± 6.4	0.11
Volume (ml)	1.9 (1.5–2.6)	2.0 (1.8–2.6)	0.45
Postoperative adverse events	23 (39.0)	71 (24.1)	0.02*
Dizziness	11 (18.6)	34 (11.5)	0.13
Delirium	6 (10.2)	21 (7.1)	0.42
Headache	8 (13.6)	27 (9.2)	0.30
PONV	5 (8.5)	18 (6.1)	0.50
Muscle weakness	2 (3.4)	5 (1.7)	0.73
Other severe unexpected events	2 (3.4)	11 (3.7)	1.00

N, number of patients; BMI, body mass index; AVM, arteriovenous malformation; DAVF, dural arteriovenous fistula; CCF, carotid-cavernous fistula, BIS, bispectral index.

* *P < 0.05*, **P < 0.01.

On univariable analysis, TCR cases more likely occurred in DAVF (78.0% vs. 19.7%, *P* < *0.01*) and middle meningeal artery embolisation (72.9% vs. 15.9%, *P* < *0.01*). The composite incidence of postoperative adverse events in group T was significantly higher than that in the group N (39.0% vs. 24.1%, *P < 0.05*). However, the incidence of single postoperative adverse events were quite similar in two groups.

Multivariate logistic regression analysis (Table 2) identified that the independent risk factors of TCR were DAVF [OR = 4.12; 95% CI (1.83–10.65); *P < 0.01*], and middle meningeal artery embolization [OR = 3.90; 95% CI (1.58–9.63); *P < 0.01*]. The results also showed that there was no significant difference in the incidence of TCR among BIS values *(P > 0.05)*.

**Table 2:** Univariate and multivariate logistic regression analysis about TCR episode.

Predictor	Univariable		Multivariable	
	OR (95% CI)	*P*-value	OR (95% CI)	*P*-value
Age (yr)	1.01 (0.99, 1.03)	0.25	–	–
Gender (M)	0.66 (0.36, 1.20)	0.17	–	–
Protopathy				
AVM	0.08 (0.03, 0.20)	<0.01**^a^	0.62 (0.16, 2.48)	0.50
MultivariableDAVF	14.46 (7.33, 28.52)	<0.01**^a^	4.12 (1.83, 10.65)	<0.01**
Supplying vessels				
Cerebral artery	0.08 (0.04, 0.18)	<0.01**^a^	0.60 (0.17, 2.08)	0.42
Middle meningeal artery	14.20 (7.38, 27.25)	<0.01**^a^	3.90 (1.58, 9.63)	<0.01**
Bradycardia	1.03 (0.45, 2.33)	0.94		
BIS value				
<40	1.33 (0.90, 1.96)	0.15	–	–
40–50	1.15 (0.66, 2.01)	0.63	–	–
>50	1.31 (0.72, 2.38)	0.38	–	–

AVM, arteriovenous malformation; DAVF, dural arteriovenous fistula; CCF, carotid-cavernous fistula; BIS, bispectral index; OR, odds ratio; CI, confidence interval.

^a^ Analyzed using multivariate analysis.

**P < 0.01.

**Figure 2:**
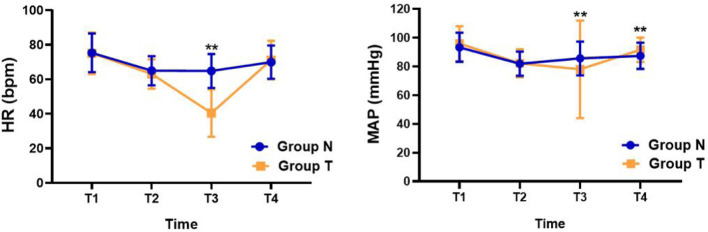
Changes in heart rate and mean arterial pressure during the study. Data are shown for control and TCR groups at 4 time points during the study: T1, 5 min before anesthetic induction; T2, 1 min before DMSO/Onyx injection; T3, at the moment of DMSO/Onyx injection; T4, at the end of the operation. *P < 0.05, **P < 0.01, TCR group vs. the control group at certain time points. Abbreviations: HR, heart rate; MAP, mean arterial pressure.

As shown in Figure 2, during DMSO/Onyx injection, HR was much slower (40.4 ± 13.8 bpm vs. 65.4 ± 10.7 bpm, *P < 0.01*), and MAP was lower in group T (78.0 ± 33.9 mmHg vs. 86.2 ± 12.7 mmHg, *P < 0.01*) than that in group N. However, the blood pressure shows either decrease or increase in group T during TCR episode in Table 3. 37 patients presented with hypotension and 22 patients with hypertension. Among these patients, middle meningeal artery embolization induced TCR was more closely associated with hypotension *(P < 0.05)*.

**Table 3:** Description of patients with significant hemodynamic fluctuations.

	Total	DAVF	Other protopathy	*P*-value	Middle meningeal artery	Other vessels	*P*-value
TCR	59	46 (78.0)	13 (22.0)	<0.01**	43 (72.9)	16 (27.1)	<0.01**
Heart arrest	5	3 (6.5)	2 (15.4)	0.65	4 (9.3)	1 (6.3)	1.00
TCR with MAP decrease	37	28 (60.9)	9 (69.2)	0.81	31 (72.1)	6 (37.5)	0.02*
TCR with MAP increase	22	18 (39.1)	4 (30.8)		12 (27.9)	10 (62.5)	

HR, heart rate; MAP, mean arterial pressure.

* *P < 0.05*, **P < 0.01.

Univariate and multivariate logistic regression analysis (Table 4) were applied to analyze postoperative adverse events. The results showed that the TCR episode [OR = 2.18; 95% CI (1.20–3.95); *P < 0.05*] and the history of preoperative intracerebral hemorrhage [OR = 1.84; 95% CI (1.13–3.00); *P < 0.05*] were associated with postoperative adverse events.

**Table 4:** Univariate and multivariate logistic regression analysis of postoperative adverse events.

Predictor	Univariable		Multivariable	
	OR (95% CI)	*P*-value	OR (95% CI)	*P*-value
Age < median (yr)	1.00 (0.99, 1.02)	0.88	–	–
Gender (M)	0.90 (0.55, 1.47)	0.67	–	–
Protopathy				
AVM	1.12 (0.67, 1.88)	0.66	–	–
DAVF	0.74 (0.46, 1.20)	0.22	–	–
CCF	1.79 (0.84, 3.81)	0.13	–	–
Intracranial tumors	0.98 (0.51, 1.90)	0.95	–	–
Supplying vessels				
Middle meningeal artery	1.60 (0.95, 2.69)	0.08	–	–
Cerebral artery	0.74 (0.46, 1.19)	0.22	–	–
Comorbidity				
Hypertension	1.39 (0.79, 2.44)	0.26	–	–
Diabetes mellitus	0.88 (0.34, 2.27)	0.79	–	–
History of cerebral hemorrhage	1.75 (1.08, 2.84)	0.02*^a^	1.84 (1.13, 3.00)	0.02*
TCR episode	2.05 (1.14, 3.70)	0.02*^a^	2.18 (1.20, 3.95)	0.01*

The postoperative adverse events (within the first 24 h after operation) were dizziness, delirium, headache, PONV (Post operative nausea and vomiting), muscle weakness, aphasia and hypopsia.

* *P < 0.05*.

^a^ Analyzed using multivariate analysis.

## Discussion

TCR is a critical cardiovascular event found in several surgical procedures on the trigeminal nerve-innervated anatomical structures. TCR can be seen during endovascular embolization and results in an intense autonomic disturbance of the heart that manifests as the sudden onset of bradycardia, hypotension, arrhythmias and even heart arrest. Sudden hemodynamic fluctuations may affect the course of surgery and patient prognosis. In this study, we demonstrated that the DAVF and middle meningeal artery embolization were independent risk factors for TCR, while the composite incidence of postoperative adverse events were significantly higher when TCR was present (39.0% vs. 24.1%). More patients experienced dizziness, headache, and delirium after surgery, which may be related to the dramatic hemodynamic fluctuations during TCR. We also found that middle meningeal artery embolization highly likely caused hypotension, implicating a more severe reflex variant under this condition.

In this study, all the TCR patients were belong to central type, however, we observed that just 37 patients experienced hypotention, but 22 patients presented with hypertention. Therefore, we suggest that central stimulant from chemical agents and distinct endovascular embolization might share the common efferent pathway for vagus activation, and the extent of vagus nerve activation might determine the alteration of blood pressure. Severe bradycardia causes hypotension when vagus nerve is profoundly activated, while relatively mild activation of vagus nerve leads to hypertension due to simultaneous stimulation on the inferior cardiovascular sympathetic nerve. The low incidence of hypotension in this study may be associated with the slower rate of ONYX injection and the greater susceptibility of sympathetic nerve stimulation. Meanwhile, continuous invasive arterial blood pressure monitoring can detect hemodynamic changes in time and immediately initiate the treatment process to prevent over-activation of the vagus nerve.

In this cohort, we found that in the TCR group, the patients with protopathy of DAVF accounted for 78.0% [OR = 4.12; 95% CI (1.83–10.65)], and the patients received middle meningeal artery embolization accounted for 72.9% [OR = 3.90; 95% CI (1.58–9.63)], which was significantly higher than that in the control group. Therefore, we conclude that the patients with DAVF or middle meningeal artery embolization are the risk factors for TCR episode during DMSO/Onyx injection. These results are consistent with the previous case reports that TCR occurs mainly in patients with DAVF during cerebrovascular interventional embolization operation (9).

DAVFs are abnormal connections between dural arteries and venous sinuses. The dura mater is innervated in part by branches of the trigeminal nerve and receives vascular supply from the meningeal artery as well as meningeal branches of the occipital artery (13). These vessels are primarily involved in the blood supply to the sensory area of the trigeminal nerve. Therefore, when the meningeal artery is stimulated, the trigeminal nerve attached to the artery is activated, resulting in a TCR. Further analysis revealed a significantly higher incidence of hypotension during middle meningeal artery embolization. It is the main supplying vessel for dura mater, and richly surrounded by trigeminal nerve sensory fibers that constitute the trigeminal neurovascular system. Stimulation of the middle meningeal artery will cause extensive excitation of the trigeminal nerve sensory endings, and then transmit the nociceptive stimulation to the cerebral center. Such stimulation signals cause the occurrence of central TCR that manifest as severe bradycardia and hypotension. To some extent, these results further support the anatomically specific triggering of TCR. Therefore, it should be noted that when operation on the middle meningeal artery with Onyx embolization in DAVF patients, anesthesiologists and neurointerventionist need to keep in mind to adopt appropriate strategies which has been recommended by Schaller and colleagues (10) to avoid occurrence of TCR.

Previous studies have revealed that Onyx do not affect hemodynamics by itself (14). However, mechanical stimulation of the trigeminal nerve during DMSO/Onyx injection is an essential inducement of TCR, and this effect is closely related to the dose and rate of injection (15). It has benn recommend that the injection rate of both DMSO and Onyx should not exceed 0.1 ml/min (16). In this study, there was no significant difference in embolization dose between the two groups, and all procedures were performed by the same experienced neurointerventionist in accordance with surgical requirements. Therefore, we do not expect a relevant difference in injection dose and injection rate and assume that this is not a relevant parameter for this study.

The occurrence of TCR is hard to predict during cerebrovascular embolization and there is no precise detection index. Currently, preventive measures for TCR mainly include the use of anticholinergic drugs to reduce vagal excitability and increase the heart rate (17). However, in some profound TCR cases, preemptive administration of anticholinergics for the prevention of TCR may be ineffective. It has been observed that bradycardia and hypotension in TCR includes both excessive activation of vagal and inhibition of adrenergic vaso-constriction after electrical stimulation of the spinal trigeminal tract and trigeminal nuclear complex (18). Atropine can only block the cholinergic fibers, yet cannot completely prevent bradycardia or hypotension. In this study, we also found that some patients often showed MAP increasing during embolization treatment, and atropine may increase the risk of hypertension while increasing the heart rate. In addition, atropine is at risk of refractory arrhythmia, so prophylactic use is not routinely recommended (19). In this study, 5 patients who suffered cardiac arrest resumed spontaneous circulation with atropine or small doses of epinephrine. Asystole as a result of severe TCR occasionally occurs during Onyx embolization, which was possibly due to the manipulation of Onyx embolization, speed and amount of DMSO/Onyx injection, enhanced vagal activity, etc. Similarly, multiple patients with HR < 40 bpm or recurrent TCR during embolization were improved with 0.5 mg atropine. We found that there was no significant difference in preoperative or before embolization HR between two groups and preembolization sinus bradycardia did not increase the risk of TCR. However, prophylactic correction of bradycardia may be beneficial for TCR high-risk patients with severe bradycardia before embolization. Recently, two case reports from Coleman et al (4) and our group (20) described that prophylactic intra-arterial injection of lidocaine was able to blunt TCR during endovascular treatment of CCF and DAVF. This approach may be become a novel strategy for TCR prevention and treatment.

Previous studies have demonstrated the depth of anesthesia is an important factor which affecting TCR episode. A meta-analysis (21) showed that lighter anesthesia (CSI > 60) were associated with a 1.2-fold increased incidence of bradycardia and a 4.5-fold increased risk of cardiac arrest than deeper anesthesia (CSI < 40). In our study, there was no significant difference in BIS value between the two groups and stratified analysis results also suggested that different depth of anesthesia had no significant influence on the occurrence of TCR, which may be related to the absence of patients with lighter anesthesia. However, this study showed that the incidence of postoperative adverse events in TCR group was significantly higher than that in the control group. Multivariate regression analysis also indicated that TCR episode was one of the independent risk factors. This may be mainly attributed to the change of intracranial pressure caused by the drastic fluctuation of intraoperative hemodynamics and abnormal embolism caused by surgical interruption.

The limitation of this study is being a single-centered study with a relatively limited sample size and potential bias in patient selection. Meanwhile, this study is a retrospective study, in which only BIS values were used as the evaluation index for anesthesia depth.

## Conclusion

In summary, this study showed that DAVF and middle meningeal artery embolization are independent risk factors for TCR during Onyx endovascular embolization. TCR increases the risk of perioperative hemodynamic fluctuations and the incidence of postoperative adverse events. Therefore, our results might guide anesthesiologists and neurointerventionist to adopt efficient and appropriate prevention and treatment strategies for TCR during cerebrovascular interventional procedures, especially when performing Onyx embolization on DAVF patients with middle meningeal artery affected.

## Data Availability

The raw data supporting the conclusions of this article will be made available by the authors, without undue reservation.
